# Prevalence and Self-Medication for Acne among Students of Health-Related Science Colleges at King Saud University in Riyadh Region Saudi Arabia

**DOI:** 10.3390/medicina59010052

**Published:** 2022-12-27

**Authors:** Ziyad Alrabiah, Azher Arafah, Muneeb U. Rehman, Salmeen D. Babelghaith, Wajid Syed, Fahad K. Alrashidi, Fahad F. Aldajaani, Musab A. Alsufayan, Mohamed N. Al Arifi

**Affiliations:** Department of Clinical Pharmacy, College of Pharmacy, King Saud University, Riyadh 11451, Saudi Arabia

**Keywords:** acne, self-medication, university students, Saudi Arabia

## Abstract

*Background and Objectives:* In Saudi Arabia, Acne vulgaris is a very predominant ailment among adolescents, especially female university students, and self-medication has become a trend to manage this condition. To determine the prevalence of Acne vulgaris among health care students and to access the scenario of its self-medication in light of students’ knowledge, attitude, and practice towards it. *Materials and Methods:* This was an observational study conducted at King Saud University, Riyadh, Kingdom of Saudi Arabia, from January 2022 to March 2022. The study was undertaken using a pre-structured questionnaire. *Results:* A total of 550 university students were recruited and the incidence of acne was observed to be 78.5% (432 out of 550) with a female predominance. A total of 56.0% (244 of 432) students used self-medications for acne without a prescription and the most used prescription drugs were topical and oral antibiotics (38.1%), followed by Isotretinoin (22.55), and topical adaplene (20.9%). Female students (*n* = 181, 63.5%) were significantly more likely to self-medicate compared to male students (*n* = 63, 42.9%, *p* ≤ 0.001). Almost 60% of medical students had proper knowledge of medication for acne. *Conclusion:* Acne vulgaris is a highly prevalent condition among university students of Saudi Arabia and use of self-medication among acne sufferers is high. Education programs should be made to raise awareness about acne and its treatment.

## 1. Introduction

Approximately 9.4% of the world’s population suffers from acne vulgaris, commonly known as acne [1]. The syndrome affects 85% of both male and female teenagers and can last well into adulthood [2]. Various studies, however, estimate prevalence in teenagers ranging from 28.9% to 91% [3,4]. A survey in Jeddah, Saudi Arabia, in 2013, aiming at examining public understanding and perceptions about acne, revealed that 64.5% of the population had acne, and 28.4% of the people believed that diet plays the most critical role in acne [5]. More recently, a study conducted in Riyadh city reported 78% acne among a study group aged between 15 and 30 years, and acne was more common in women (86.1%) than in men (69.9%; *p* = 0.001) [6]. On the other hand, the prevalence of acne among medical students in Saudi Arabia has previously been observed to be 55%. Among them, the majority (87.2%) had a perception of hormonal imbalance in its pathogenesis [7]. Another study showed that truncal acne affected 42% of female medical students, with acne scars in 60.9% of subjects [8].

Self-medication is defined as the use of non-prescribed medicines, mainly, over-the-counter (OTC) pills, to cure minor symptoms without seeking medical advice [9]. Self-medication can take several forms, such as obtaining medication without a prescription, sharing drugs with others, or using a medicine already at home [9]. Medications to treat acne and other infections are easily accessible in Saudi Arabia and can be purchased over the counter without a prescription from a medical practitioner [10]. A systematic review study mentioned that 52% of acne patients utilized some type of OTC medications at least once [6]. Cleansers were the most widely used OTC drugs [11]. In addition, a variety of factors contribute to the practice of self-medication among health care students, such as easy availability, exposure to medical settings, and pharmacological knowledge [12]. The prevalence of self-medication for acne among Pakistani medical students was 50.4% [12]. Two independent studies conducted among students of medicine and allied specialties reported that 35.8% [8] and 52.4% [11] of students practiced self-medication for acne and used OTC drugs.

There are different treatment approaches for acne. For instance, the most common form is the use of plant remedies, in combination with lactic acid [13], the application of an exfoliating and cleansing treatment more commonly known as oxybrasion [14]. All these treatment approaches work effectively on the skin, including fewer skin breakouts and lower sebum levels on the skin’s surface. Furthermore, it was found that acne was more common among students and significantly associated with gender [15]. On the other hand, earlier study also reported misconceptions and false beliefs on acne about psychological problems. In addition, poor outcomes of treatment, or neglecting the condition, increases the skin sensitivity, negative feelings, and anxiety, which would negatively affect the quality of life acne patients [16,17,18,19]. Quality of life is crucial in individuals’ lives to have a balanced and healthy lifestyle [18,19].

Despite the world health organization (WHO) support for self-medication for minor ailments, it warns against its drawbacks, such as unpleasant side effects and the emergence of multidrug resistance [12]. Self-medication for acne has been on the rise globally as well as locally. In addition, no study of the knowledge, attitude, and practice (KAP) of self-medication for acne among university students in Saudi Arabia has been done to date. So, our study focuses on determining the prevalence of acne and its self-medication, as well as to assessing the KAP of acne self-medication among King Saud university students in the Riyadh region of Saudi Arabia.

## 2. Methods

### 2.1. Study Design

This was a cross-sectional internet-based study conducted between January 2022 and March 2022 among students enrolled in King Saud University’s Colleges of Health, Pharmacy, Dentistry, Nursing, and Applied Medical Sciences. Written informed consent was taken from the participants before their inclusion in the survey. The data collection was carried out using a social medial platform. For the purpose of data collection, Google forms were used, and an electronic link was generated and sent to the targeted participants; for this purpose, the snowball technique was used, where one participant refers to another participant. The data collection was followed by the convenience sampling method.

### 2.2. Sample Size

A total of 550 university students of different streams were recruited for the study. According to prior estimates, the prevalence rate of acne among medical students was 55% [20]. The total sample size came to be three hundred and eighty (*n* = 380) using the following equation:

*n* = z^2^ × p × q/d^2^

where *n* is the minimum sample size, z is the constant (1.96), p is the prevalence of acne among medical students, (0.55), q is (1 − p), Z is the standard normal deviation of 1.96 corresponding to the 95% confidence interval, and d is the desired degree of accuracy.

### 2.3. Pre-Structured Questionnaire

A paper-based self-administered questionnaire was used to collect data, designed by a team of professionals (one researcher or pharmacist and professor) who have experience in this regard. The questionnaire utilized in this study was created after thoroughly evaluating the literature on healthcare students’ knowledge, prevalence, and self-medication of acne [21]. The first component of the questionnaire included a demographic section and questions concerning the site of acne, type of self-medications, over-the-counter acne treatments, the reason for self-medications, and information sources. The questionnaire’s second section included questions to grasp students’ knowledge, attitude, and practices towards self-medication. It had a six-question instrument to assess the student’s knowledge towards self-medication for acne, which included the dosage of the drug, mode of action, adverse drug reaction, precautions for use, contraindications, and complications. It also contained four and three questions used to assess the attitude and practices of students towards self-medication for acne, respectively.

A pilot study was carried out among a randomly chosen small sample of health care students (*n* = 30) to ensure the questionnaires’ readability and simplicity of administration. The final analysis did not incorporate the pilot study’s findings. With a Cronbach’s Alpha score of 0.81, the reliability of the questionnaire was determined, suggesting its suitability for the study.

### 2.4. Calculation of Knowledge Score

According to previous research, knowledge scores were calculated for each scale’s items by categorizing the participants’ responses as correct or incorrect [22,23,24,25,26]. In all knowledge questions about acne self-medication, the answer “Yes” received a score of 1, while the incorrect response received a score of 0. (No). Adding one point to each accurate response and zero to each incorrect response resulted in the knowledge score. Each student received a final score that varied from 0 to 6.

### 2.5. Statistical Analysis

**Figure 1:**
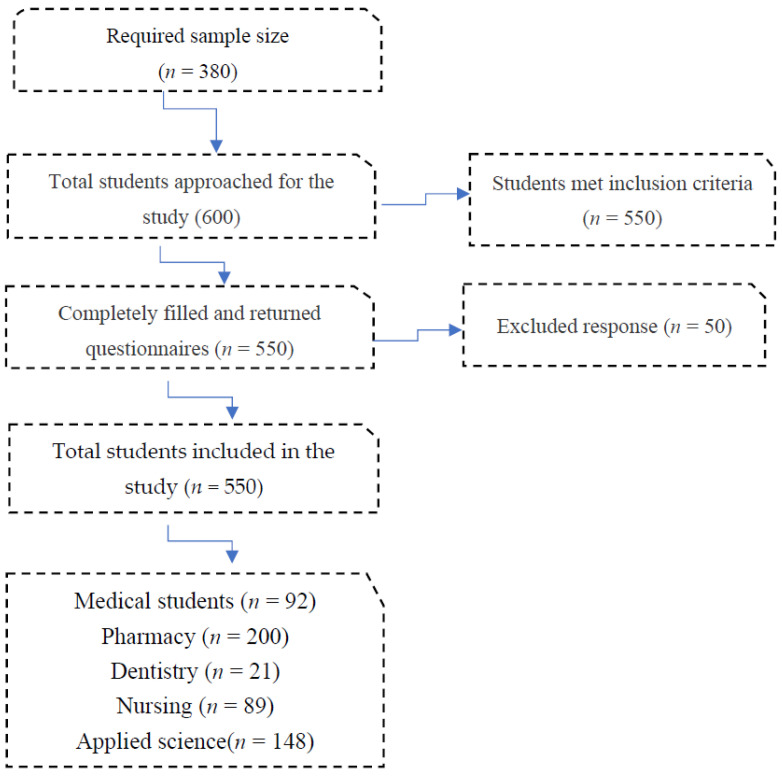
Flowchart of the responses.

The data were presented in the form of frequencies and percentages. The Statistical Package for Social Sciences (SPSS) version 26.0 (IBM Corp., Armonk, NY, USA) was used to analyze the data. The Chi-square test was used for categorical variables analysis. Kruskal Wallis and Mann–Whitney tests were used for continuous variables analysis whenever applied. A *p*-value of ≤0.05 indicated statistical significance. The study required the participation of 380 students, but we approached 600 students to avoid sample bias and there were 50 students excluded, due to mismatching of the inclusion criteria. Overall, included responses in this study are 550, giving a response rate of 91.6% (Figure 1).

## 3. Results

### 3.1. Characteristics of Study Subjects

A total of 550 students who successfully filled in the questionnaire were enrolled in the study. A total of 63.5% (349 of 550) were females, and 36.5% (201 of 550) were males. Most of the enrolled students (86.7; 477 of 550) were from public sector universities. The majority of the students were in the first year of their Bachelor’s degree course (31.5%), followed by final-year students (25.5%). Pharmacy students showed maximum participation (36.4%), followed by students of applied sciences (26.9%), with the college of dentistry having the lowest percent of responses (3.8%). The prevalence of acne in our study group was found to be 78.6% (432 of 550). Table 1 provides the detailed socio-demographic information on enrolled students in health sciences.

### 3.2. Characteristics of Acne Patients

The prevalence of acne was found to be 78.6% (432 out of 550). A total of 285 female and 147 male students suffered from acne. (Table 1) Female students had a significantly higher prevalence of acne compared to male students (66% vs. 34%, respectively, *p* = 0.023). Nevertheless, no significant association of age, educational level, and the stream were found with the presence of acne among university students.

### 3.3. Site of Acne

Table 2 shows the locations of acne lesions. About 15 (3.5%) students did not mention the location of their acne lesions. The acne was found predominantly on the face in 38.9% of students. The second predominant location was “face and back” in 24.8% of students followed by “face, chest, and back” in 19.2% of students.

### 3.4. Self-Medication for Acne

A total of 56.4% (244 of 432) students have previously used self-medications for acne without a prescription. The most frequently used drugs were topical and oral antibiotics (38.1%), followed by Isotretinoin (22.55) and topical adaplene (20.9%) as shown in Table 3. In addition, cleansers were the most commonly used over-the-counter treatments (44.3%), followed by leave-on products (13.9%) and mechanical therapies (11.1%) *(*Table 4).

Female students (*n* = 181, 63.5%) were significantly more likely to self-medicate higher than male students (*n* = 63, 42.9%, *p* ≤ 0.001). In addition, dentist students were more likely to practice self-medication compared to other students (*n* = 15, 88.2%, *p* = 0.002). 

The majority of the students (20.1%) attributed “easy accessibility of medicines” as the reason for self-medication. As per the filled questionnaire, the other main reasons for self-medication were “insufficient time” and “mild disease severity”, respectively, as shown in Table 5. The most common information resources were the internet (31.6%), friends (19.3%), and decision-making/prescriptions written for others (both 16%) as shown in Table 6.

This study assessed the knowledge of students about self-medication, which is presented in Table 7. More than half of the students knew the dosage of the drugs they were taking (52.5%). In total, 55.7% of students were well aware of the mode of action and adverse drug reactions of the drugs taken. About 56% of students knew the precautionary measures to be taken. In addition, about 53% of students were aware of the contraindications of over the counter. In total, 57.1% were aware of complications/side effects of medicines.

### 3.5. Knowledge Score of Students towards Self-Medication

However, the total mean score for knowledge was 3.3 ± 2.1. No statistical significance was observed between demographic variables and knowledge score (Table 8).

### 3.6. Students’ Attitude and Practice towards Self-Medication

Questions accessing the attitude and practice towards self-medication are contained in Table 8. Most students agreed that self-medication is a form of self-care (73.0%). About two-third of students said that they would give advice to friends for self-medication. Only 58.2% of students were of the opinion that they would consult a dermatologist before starting treatment. (Table 9). 

The last three questions contained in Table 8 were used to assess the practice towards self-medication. Most students (86.9%) agreed on reading label instructions before using medications. Most students (70.1%) reported that they read the expiry date before using medicines. The availability of self-medications at home were reported by 72.1% of students. (Table 9).

## 4. Discussion

In our study, the prevalence of acne was observed to be 78.5%. A study of available data on the incidence of acne in the Saudi people and adolescents reveals a significant prevalence rate among the Saudi population. Menon et al. in their cross-sectional study, found the prevalence of acne to be 55.0% among university students [20]. As per previous independent studies, the prevalence of acne in the Saudi population was 64.5% and 78.0%, respectively [5,6]. Similarly, a study carried out among Saudi medical students by Alajlan et al. observed a prevalence of 55.5% [7]. Another study reported that 83.4% of female Saudi students had acne [8]. A study performed by Deyab et al. found a prevalence rate of 56.0% among students of Al Majmaah University, Saudi Arabia [21].

In our survey, female students reported a significantly higher prevalence of acne than male students (66% vs. 34%). This was in comparison with a similar study done in Saudi Arabia which revealed that the prevalence of acne among male and female students was 62.5% and 51.8%, respectively [21]. The prevalence rate among high school students in Jazan province, in the south of KSA, was 71% in females compared to 60% in males [27].

In our study, the face was the most common area of the body to be affected by acne, followed by the back and chest, as previously reported [6,12]. As per the study done by Allayali et al., faces were most commonly affected by acne [3].

Self-medication for acne is a rampant habit among university students, especially medical students. In our study, 56.0% (244 of 432) of students suffering from acne practiced self-medication. Alshehri et al., in their study, reported that 52.4% of university students suffering from acne used OTC medications [11]. Similar to our findings, Karamata et al. observed 59.2% of students practicing self-medication for acne [28]. In this study, self-medication was found to be significantly more common among females (*p* = 0.001). Similar findings have been ascertained by some studies between gender and self-medication [11]. The rampant use of self-medication in females could be due to hormonal imbalance; social stress or family history [28]. A study done in Brazil showed a significant association between acne vulgaris and stress [29], linking with a study by Al Mashat et al. according to which 58.4% of individuals believed that stress aggravates acne vulgaris [5].

The majority of students (38.1%) in our study used topical and oral antibiotics for acne treatment, especially topical antibiotics. In a previous study carried out in Saudi Arabia, it was reported that only 11.4% of students used topical and oral antibiotics for their acne treatment [11]. The higher frequency of topical antibiotic use in our study group hints towards the easy availability of topical antibiotics in Saudi pharmacist shops which will in the long run create havoc in society. Our study observed 22.5% of students using isotretinoin as self-medication for acne, which is close to the findings of Alanazi et al., who reported that 19.2% of the acne-affected population use isotretinoin [6]. However, as per a previous study conducted in Saudi Arabia, 44.2% of the public used isotretinoin as their first choice for mild acne, which is against recommended guidelines [30]. Nevertheless, inappropriate use of antibiotics may increase the risk of adverse effects or antimicrobial resistance even when applied topically [28]. As a result, health authorities should act decisively to put into effect the 1978 statute that the Ministry of Health passed to stop the selling of antibiotics without a prescription. Additionally, they should encourage the creation of national standards for antibiotic use and prescription that are supported by research. In order to stop the local and worldwide rise of antibiotic resistance, health awareness programs against antibiotic abuse should be launched among pharmacists, prescribers, and the general public [29].

OTC acne therapies are divided into 6 main categories: cleansers, leave-on products, mechanical treatments, essential oils, vitamins, home remedies, and herbal treatments. For some patients with minor acne, OTC medications can be useful [11]. In this study, cleansers were the most common OTC treatments (44.3%). It has been established that females use self-treatment products more often than male patients, such as cleansers, acne camouflage or cover-up, and facial masks [11].

In our study, the most common reason stated behind self-medication was insufficient time followed by easy accessibility and minor disease severity. According to research conducted in Pakistan, the most common reasons for self-medication were mildness of disease and easy accessibility [12]. It is conceivable that medical students in the study group have a strict and vast curriculum, so they always run out of time, and they prefer self-treatment. The students of medicine and allied branches have easy access to medicine compared to the students of other branches like social sciences, earth sciences, and mathematical sciences. Due to the mild severity of the disease and since acne is not a life-consuming disease, the seriousness to follow a treatment protocol is lacking, prompting individuals to practice self-medication. 

As per our survey, the most common source of information for self-medication was the social internet (31.6%). In this modern era, the internet has revolutionized the world of information technology and many previous reports back the concept of the internet revolution behind the information or knowledge source in Saudi Arabia [6,7,11,31,32]. According to few studies, the most common source of knowledge regarding the self-treatment and management of acne was friends [12,33]. Although the beneficial use of the internet cannot be denied, the rampant use of the internet for practicing medicine is illegal and is giving quacks a chance to creep into the medical profession. So, the masses in general and medical students in particular should be discouraged from using the internet for medical help and should rather be encouraged to seek knowledge from the authentic sources such as published medical literature and skilled mentors.

More than half of the students had appropriate knowledge about the dosage of the drug; mode of action; adverse drug reaction; precautions for use; contraindication and associated complications, which means that the enrolled medical students had an average knowledge of self-medications. As per a few previous studies, nearly 35.0% of the students did not know the potential side effects of the self-prescribed medicines, hinting at their poor knowledge of self-medications [34,35]. Despite the little knowledge about self-medication, the attitude of our study participants was positive, which is very dangerous, as the drugs taken could not always cure the diseases but could have some deleterious health effects which might manifest later during the lifetime. In our study, 73.0% of students believe self-medication is part of self-care and 59.0% encouraged friends and family to self-medicate. Our observation is in line with the independent studies done by Tameez-Ud-Din et al. and Raikar et al. wherein almost 60.0% of students used self-medication for acne and recommended it to their friends [12,36].

Our study found that almost 70.0% of students had an attitude of reading leaflets/label instructions and expiry dates on medicines, indicating that during pharmacology and clinical training, students are trained to read the labels before using medications. This study assessed self-medication used for acne by undergraduate pharmacy. About 38% in our study used topical and oral antibiotics for acne. Even when applied topically, antibiotics may increase resistance. Therefore, it is crucial for undergraduate pharmacy students to be aware of self-medication in order to avoid negative drug reactions and antimicrobial resistance. The future of rational prescribing will be improved by awareness of self-medication and its effects. Future studies are required to fill up knowledge gaps and dispel myths about acne vulgaris among children and teenagers.

### Limitations

This study has some limitations. First, this study involved only university students, and as they are often expected to be more informed, extrapolating the findings to the wider population may not be trustworthy. Second, a lack of understanding of the clinical criteria of acne may have contributed to a little lower prevalence of acne and self-medication than anticipated. Third, the design of this study was cross-sectional, and limited to a single university in Saudi Arabia, which cannot be generalized to the whole population. Therefore, extrapolating the results of this study to other areas could not give an accurate picture. Fourth, this study did not include topics like the specific, comprehensive contents, effects, and likely side effects of OTC medications.

## 5. Conclusions

Acne is prevalent among adolescents, predominantly female health-related science college students, in King Saud University, Riyadh, Saudi Arabia. Due to the mild pathogenicity, its self-medication is rampant. In addition, female students used self-medication more than male students. Acne vulgaris, its treatment, and complications need to be better understood, and those undertreated must be provided with proper management plans using the stepwise approach. Healthcare institutions should only stress the use of drugs such as antibiotics by a registered medical practitioner. In addition, awareness should be spread regarding the strict dispensing of antibiotics so as to do away with multi drug resistance in future.

## Figures and Tables

**Table 1:** Demographic and pathological characteristics of Saudi health care students.

Variables	Freequency (*n*) (550)	Percentages (%)
Gender Male Female	201 349	36.5 63.5
Age groups 18–26 27–30 31–35 >36	518 21 08 03	94.2 3.8 1.5 0.5
Education level First year Second year Third year Fourth year Fifth year Final year	173 98 75 40 24 140	31.5 17.8 13.6 7.3 4.4 25.5
College type Medicine Pharmacy Dentistry Nursing Applied sciences	92 200 21 89 148	16.7 36.4 3.8 16.2 26.9
Development of acne No Yes	118 432	21.4 78.6

**Table 2:** Site of acne lesions (*n* = 432).

Site	Freequency (*n*)	Percentages (%)
Back	29	6.7
Chest	4	0.9
Check and back	8	1.9
Face	168	38.9
Face and back	107	24.8
Face and chest	18	4.2
Face, chest, and back	83	19.2
Missing	15	3.5

**Table 3:** Type of medication (*n* = 244).

Type of Medication	Freequency (*n*)	Percentages (%)
Topical and oral antibiotic	93	38.1
Topical adaplene	51	20.9
Topical azelaic acid	42	17.2
Isotretinoin	55	22.5
Anti-androgens (spironolactone)	03	1.2

**Table 4:** Common types of over-the-counter acne treatments (*n* = 244).

Type	Freequency (*n*)	Percentages (%)
Cleansers	108	44.3
Leave-on products	34	13.9
Mechanical treatment	27	11.1
Essential oils	19	7.8
Vitamins	21	8.6
Home remedies and herbal	18	7.4
Missing data	17	7.0

**Table 5:** Reason for self-medication (*n* = 244).

Reasons	Freequency (*n*)	Percentages (%)
Disease severity is mild	48	19.7
Easy accessibility	49	20.1
Know the treatment prescribed previously	29	11.9
Insufficient time	50	20.5
Pharmacology knowledge	32	13.1
Feeling embarrassment when discussing symptoms	8	3.3
Not interested in involving faculty	6	2.5
Missing	22	9.0

**Table 6:** Sources of information (*n* = 244).

Source of Information	Freequency (*n*)	Percentages (%)
Friends	47	19.3
Prescriptions written for others	39	16.0
Decision-making	39	16.0
Drug advertisements	18	7.4
Books	24	9.8
Internet	77	31.6

**Table 7:** Students’ knowledge towards self-medication (*n* = 244).

Variables	Yes *n* (%)	No *n* (%)
Dosage of the drug	128 (52.5)	116 (47.5)
Mode of action	136 (55.7)	108 (44.3)
Adverse drug reaction	136 (55.7)	108 (44.3)
Precautions for use	137 (56.1)	107 (43.9)
Contraindication	128 (52.5)	116 (47.5)
Complication	139 (57.0)	105 (43.0)

**Table 8:** Students’ knowledge score towards self-medication (*n* = 244).

Variables	Mean ± SD	*p* Value
Total	3.3 ± 2.1	
Gender Male Female	3.2 ± 2.2 3.3 ± 2.1	0.859
Age group 18–26 27–30 31–35 > 36	3.3 ± 2.1 4.2 ± 1.6 2.0 ± 1.6 4.5 ± 2.1	0.236
College type Medicine Pharmacy Dentistry Nursing Other	3.5 ± 2.0 3.0 ± 2.4 3.4 ± 1.6 3.5 ± 2.0 3.4 ± 2.0	0.753

**Table 9:** Students’ attitude and practice towards self-medication (*n* = 244).

Attitude and Practice Parameters	Yes *n* (%)	No *n* (%)
Self-medication is a component of self-care	178 (73.0)	66 (27.0)
Encourage friends and family to self-medicate	144 (59.0)	100 (41.0)
Does acne require a dermatologist’s consultation?	142 (58.2)	102 (41.8)
Follow-up for acne is important?	201 (82.4)	43 (17.6)
How often do you read the directions on a drug label?	168 (68.9)	76 (31.1)
When purchasing a drug, do you check its expiration date?	171 (70.1)	73 (29.9)
Are the medications available at home/hostel at all times?	176 (72.1)	68 (27.9)

## Data Availability

The data used for this study will be available from the correspondence, upon the request.

## References

[1] Sachdeva M., Tan J., Lim J., Kim M., Nadeem I., Bismil R. (2021). The prevalence, risk factors, and psychosocial impacts of acne vulgaris in medical students: A literature review. Int. J. Dermatol..

[2] Lynn D.D., Umari T., Dunnick C.A., Dellavalle R.P. (2016). The epidemiology of acne vulgaris in late adolescence. Adolesc. Health Med. Ther..

[3] Allayali A.Z., Asseri B.N., AlNodali N.I., Alhunaki R.N.M., Algoblan S.F.G. (2017). Assessment of prevalence, knowledge, attitude, and psychosocial impact of acne vulgaris among medical students in Saudi Arabia. J. Clin. Exp. Dermatol. Res..

[4] Tayel K., Attia M., Agamia N., Fadl N. (2020). Acne vulgaris: Prevalence, severity, and impact on quality of life and self-esteem among Egyptian adolescents. J. Egypt. Public Health Assoc..

[5] Al Mashat S., Al Sharif N., Zimmo S. (2013). Acne awareness and perception among population in Jeddah, Saudi Arabia. J. Saudi Soc. Dermatol. Dermatol. Surg..

[6] Alanazi T.M., Alajroush W., Alharthi R.M., Alshalhoub M.Z., Alshehri M.A. (2020). Prevalence of acne vulgaris, its contributing factors, and treatment satisfaction among the Saudi population in Riyadh, Saudi Arabia: A cross-sectional study. J. Dermatol. Dermatol. Surg..

[7] Alajlan A., Al Turki Y.A., AlHazzani Y., Alhowaish N., AlEid N., Alhozaimi Z., Al Saleh W., Yahya A.B., Alkriadees Y., Alsuwaidan S. (2017). Prevalence, level of knowledge and lifestyle association with acne vulgaris among medical students. J. Dermatol. Dermatol. Surg..

[8] Zari S., Turkistani A. (2017). Acne vulgaris in Jeddah medical students: Prevalence, severity, self-report, and treatment practices. J. Cosmet. Dermatol. Sci. Appl..

[9] Alfadly S., Ballaswad M.R., Amra A.S., Alghadeer S.M., Wajid S., Al-Arifi M.N., Babelghaith S.D. (2017). Self-medication with antibiotic amongst adults attending community pharmacies in Mukalla district, Yemen. Lat. Am. J. Pharm..

[10] Albatti T.H., Alawwad S., Aldueb R., Alhoqail R., Almutairi R. (2017). The self medication use among adolescents aged between 13–18 years old; Prevalence and behavior, Riyadh–Kingdom of Saudi Arabia, from 2014–2015. Int. J. Pediatr. Adolesc. Med..

[11] Alshehri M.D., Almutairi A.T., Alomran A.M., Alrashed B.A., Kaliyadan F. (2017). Over-the-counter and prescription medications for acne: A cross-sectional survey in a sample of university students in Saudi Arabia. Indian Dermatol. Online J..

[12] Tameez-Ud-Din A., Malik I.J., Bhatti A.A., Din AT U., Sadiq A., Khan M.T., Chaudhary N.A., Arshad D. (2019). Assessment of Knowledge, Attitude, and Practices Regarding Self-medication for Acne Among Medical Students. Cureus.

[13] Chilicka K., Rogowska A.M., Rusztowicz M., Szyguła R., Yanakieva A., Asanova B., Wilczyński S. (2022). The Effects of Green Tea (*Camellia sinensis*), Bamboo Extract (*Bambusa vulgaris*) and Lactic Acid on Sebum Production in Young Women with Acne Vulgaris Using Sonophoresis Treatment. Healthcare.

[14] Chilicka K., Rogowska A.M., Szyguła R., Rusztowicz M., Nowicka D. (2022). Efficacy of Oxybrasion in the Treatment of Acne Vulgaris: A Preliminary Report. J. Clin. Med..

[15] Ismail K.H., Mohammed-Ali K.B. (2012). Quality of life in patients with acne in Erbil city. Health Qual. Life Outcomes.

[16] Al Robaee A.A. (2005). Prevalence, knowledge, beliefs and psychosocial impact of acne in University students in Central Saudi Arabia. Saudi Med. J..

[17] Samreen S., Siddiqui N.A., Mothana R.A. (2020). Prevalence of anxiety and associated factors among pharmacy students in Saudi Arabia: A Cross-Sectional Study. BioMed Res. Int..

[18] Wajid S., Menaka M., Yamasani V.V. (2021). Assessment of Health-related Quality of Life among Diabetic Out patients at Warangal Region Telangana India-A Cross-sectional Study. Asian J. Pharm..

[19] Syed W., Menaka M., Parimalakrishnan S., Yamasani V.V. (2022). Evaluation of the association between social determinants and health-related quality of life among diabetic patients attending an outpatient clinic in the Warangal region, Telangana, India. J. Diabetol..

[20] Menon C., Gipson K., Bowe W.P., Hoffstad O.J., Margolis D.J. (2008). Validity of Subject Self-Report for Acne. Dermatology.

[21] Deyab A.A., Faraz A., Abdelrahim S.A., AbdulRahman B.A., Alfaleh Y., Almutairi K.A.E. (2020). Prevalence, Awareness and Psychological Impact of Acne Vulgaris among University Students. J. Res. Med. Dent. Sci..

[22] Arafah A., Rehman M.U., Syed W., Babelghaith S.D., Alwhaibi A., Al Arifi M.N. (2022). Knowledge, Attitude and Perception of Pharmacy Students towards Pharmacogenomics and Genetics: An Observational Study from King Saud University. Genes.

[23] Syed W., Alsadoun A., Bashatah A.S., Al-Rawi M.B.A., Siddiqui N. (2022). Assessment of the knowledge beliefs and associated factors among Saudi adults towards blood donation in Saudi Arabia. Hematology.

[24] Syed W., Iqbal A., Siddiqui N.A., Mothana R.A., Noman O. (2022). Attitudes and Associated Demographic Factors Contributing towards the Abuse of Illicit Drugs: A Cross-Sectional Study from Health Care Students in Saudi Arabia. Medicina.

[25] Syed W., Alharbi M.K., Samarkandi O.A., Alsadoun A., Al-Rawi M.B.A., Iqbal A., Samreen S. (2022). Evaluation of Knowledge, Awareness, and Factors Associated with Diabetes: A Cross-Sectional Community-Based Study. Int. J. Endocrinol..

[26] Samreen S., Sales I., Bawazeer G., Wajid S., Mahmoud M.A., Aljohani M.A. (2021). Assessment of Beliefs, Behaviors, and Opinions About Blood Donation in Telangana, India-A Cross Sectional Community-Based Study. Front. Public Health.

[27] Bajawi S., Salih S., Mahfouz M.S., Bajawi N., Asiri B. (2016). Acne vulgaris awareness and impact on quality of life and psychological status of adolescent school children inJazan, Saudi Arabia. Int. J. Sci. Basic Appl. Res..

[28] Karamata V.V., Gandhi A.M., Patel P.P., Desai M.K. (2017). Self-medication for Acne among Undergraduate Medical Students. Indian J. Dermatol..

[29] Alghadeer S., Aljuaydi K., Babelghaith S., Alhammad A., Alarifi M.N. (2018). Self-medication with antibiotics in Saudi Arabia. Saudi Pharm. J..

[30] Younis N.S., Al-Harbi N.Y. (2019). Public Understanding and Awareness of Isotretinoin Use and Safety in Al Ahsa, Eastern Saudi Arabia. Ther. Innov. Regul. Sci..

[31] Wajid S., Samreen S., Sales I., Bawazeer G., Mahmoud M.A., Aljohani M.A. (2022). What Has Changed in the Behaviors of the Public After the COVID-19 Pandemic? A Cross-Sectional Study From the Saudi Community Perspective. Front. Public Health.

[32] Mahmoud M.A., Wajid S., Naqvi A.A., Samreen S., Althagfan S.S., Al-Worafi Y. (2020). Self-medication with antibiotics: A cross-sectional community-based study. Lat. Am. J. Pharm..

[33] Patil S.B., Vardhamane S.H., Patil B.V., Santoshkumar J., Binjawadgi A.S., Kanaki A.R. (2014). Self-medication practice and perceptions among undergraduate medical students: A cross-sectional study. J. Clin. Diagn. Res..

[34] Wajid S., Al-Arifi M., Al Nomay H., Al Mousa Y.N., Babelghaith S.D. (2015). Knowledge and perception of community pharmacists’ towards generic medicines in Saudi Arabia. Biomed Res..

[35] Aljadhey H., Assiri G.A., Mahmoud M.A., Al-Aqeel S., Murray M. (2015). Self-medication in Central Saudi Arabia. Community pharmacy consumers’ perspectives. Saudi Med. J..

[36] Raikar D.R., Manthale N.S. (2018). A cross sectional study of self-medication for acne among undergraduate medical students. Int. J. Res. Dermatol..

